# Construction of a strawberry breeding core collection to capture and exploit genetic variation

**DOI:** 10.1186/s12864-023-09824-1

**Published:** 2023-12-05

**Authors:** T. Koorevaar, J. H. Willemsen, R. G. F. Visser, P. Arens, C. Maliepaard

**Affiliations:** 1 Wageningen University and Research Plant Breeding, Wageningen, The Netherlands; 2 Fresh Forward Breeding B.V., Huissen, The Netherlands

**Keywords:** Genetic diversity, Core collection, Haplotype reference panel, Pedigree-genomic-based relationships, Plant breeding program

## Abstract

**Background:**

Genetic diversity is crucial for the success of plant breeding programs and core collections are important resources to capture this diversity. Many core collections have already been constructed by gene banks, whose main goal is to obtain a panel of a limited number of genotypes to simplify management practices and to improve shareability while retaining as much diversity as possible. However, as gene banks have a different composition and goal than plant breeding programs, constructing a core collection for a plant breeding program should consider different aspects.

**Results:**

In this study, we present a novel approach for constructing a core collection by integrating both genomic and pedigree information to maximize the representation of the breeding germplasm in a minimum subset of genotypes while accounting for future genetic variation within a strawberry breeding program. Our stepwise approach starts with selecting the most important crossing parents of advanced selections and genotypes included for specific traits, to represent also future genetic variation. We then use pedigree-genomic-based relationship coefficients combined with the ‘accession to nearest entry’ criterion to complement the core collection and maximize its representativeness of the current breeding program. Combined pedigree-genomic-based relationship coefficients allow for accurate relationship estimation without the need to genotype every individual in the breeding program.

**Conclusions:**

This stepwise construction of a core collection in a strawberry breeding program can be applied in other plant breeding programs to construct core collections for various purposes.

**Supplementary Information:**

The online version contains supplementary material available at 10.1186/s12864-023-09824-1.

## Background

In crops, core collections are used to conserve wide genetic variation in a minimal subset of genotypes because a smaller subset is easier to manage and share. In the past, these core collections were selected based on geographic origin and phenotypes [1, 2]. However, the genotyping revolution has allowed gene banks to genotype many individuals in their collections, providing insights into the structure of their populations and enabling the creation of comprehensive core collections in various crops, for example in barley and strawberry [3, 4]. Core collections can be designed to reflect different allelic or phenotypic distributions, geographic regions, time periods or breeding goals. Depending on their design goals, core collections can be used for different goals such as to evaluate various traits across multi locations, for the selection of genotypes for a genome-wide association study or for the selection of genotypes for a haplotype reference panel in imputation-based genotyping methods [5–7].

Imputation methods that utilize a haplotype reference panel can be used to obtain highly accurate genotyping data without the need for high-coverage whole genome sequencing (WGS). This means that lower cost reduced genomic information genotyping by sequencing (GBS) techniques can be used while still the accuracy of WGS can be reached [7–10]. The rationale behind this method is as follows: Any two genotypes can share common haplotypes, even if they seem unrelated. Subsequently, if a study genotype undergoes reduced genomic information retrieval via GBS, its incomplete genomic data can be used to identify haplotypes that are shared between this study genotype and a reference panel of haplotypes. Then, common reference haplotypes can be used to impute the missing data of the study genotype resulting in accurate genomic information [10–12]. For this, a core collection of genotypes from which haplotypes can be obtained is needed. A good haplotype reference panel reference panel represents the entire breeding germplasm as much as possible, since missing haplotypes limit imputation accuracy, *i.e.,* a shared haplotype can only be found if it is present in the haplotype reference panel [7, 8, 12]. Therefore, a core collection of genotypes that represent the haplotype diversity of the breeding germplasm is needed to obtain an optimal haplotype reference panel.

In an ideal case, this representative core collection in a plant breeding program is based on a minimum subset of genotypes which captures most or all representative genetic variation. However, selecting this set of genotypes is a non-trivial task.

In the plant breeding domain, core collections are usually constructed and maintained by gene banks to represent and conserve the genetic diversity of their collection. Most gene banks base their core collections on landraces, wild relatives and sometimes progenitors of a certain crop and aim to maximize genetic diversity. Plant breeding programs, however, consist of varieties and selections in various stages of the phenotypic selection process and targeted to select beneficial trait alleles. These plant breeding programs are often accompanied by a pre-breeding program which in addition contains progenitors and various kinds of specific genotypes (maintained for specific traits, *e.g.*, disease resistances in wild relatives). Hence, plant breeding programs (including their pre-breeding program) have a different structure and aim compared to gene bank collections.

Core collection strategies come in different types, which have already been defined in the context of gene banks [6]. For example, a CC-I core collection ensures that all genotypes in the whole collection are maximally represented in the core collection where each genotype in the whole collection is represented by an individual in the core collection that is most similar to it. This type of core collection is used for maximizing genetic diversity. Another type of core collection (CC-X) aims to represent the extremes of the whole collection. A core collection of this type contains genotypes that are as different as possible from each other resulting in a core collection that represents the maximum range of variation.

In the context of a breeding program, in principle, a CC-I core collection gives the best representation of the whole breeding program. We also want to avoid redundancies and overrepresentation of certain genetic variation (*i.e.,* only include a single individual of a full-sib family) to effectively use the available resources [6, 13]. A CC-X core collection does not fit our objective, we do not want the maximum range of variation but the maximum representation of the variation.

To obtain core collections, distance-based optimization criteria have been defined [6]. For instance, a CC-I collection is obtained by minimizing the average distance between each genotype in the whole collection and the nearest entry in the core collection (A-NE). This procedure is already implemented in the program *Core Hunter 3* [14].

A central requirement for building a core collection is that relationship coefficients among all genotypes are known: These are needed for optimizing the distance-based criteria to select the individuals for a core collection. There are two main approaches to estimate relationships among genotypes: 1) One is based on pedigree records, where average relationship coefficients are calculated which can be identified as identity-by-descent (IBD) relationship coefficients. 2) The other is based on genomic information which computes the genomic-based coefficients that can be identified as identity by state (IBS). Both genomic-based coefficients and pedigree-based relationship coefficients estimate the same relationships [15].

Several advantages suggest the use of genomic-based coefficients: these are often more accurate than the pedigree-based relationship coefficients because they even distinguish full-sibs whereas pedigree-based relationship coefficients do not [16]. Another important limitation of pedigree-based relationship coefficients is the occurrence of missing links. For instance, some genotypes may be closely related through a common ancestor not recorded in the pedigree. Such a relationship will then not be in the pedigree-based relationship coefficients.

Many plant breeding programs have pedigree records available for most genotypes, although with varying accuracy, as pedigree errors can occur. Genomic-based relationship coefficients can correct for these missing links and pedigree errors, but genomic data may be available for only a subset of genotypes. The best solution then is to combine genomic-based relationship coefficients with the pedigree-based relationship coefficients resulting in a hybrid pedigree-genomic relationship matrix, the *H* matrix. First, the pedigree-based relationship coefficients of genotyped individuals are replaced by the genomic-based relationship coefficients. Then, the rest of the pedigree-based relationship matrix is adjusted by extending the genomic-based information of the genotyped individuals to their ungenotyped relatives resulting in the genomic-pedigree-based relationship matrix *H* [17].

In this study, we propose a method for constructing a core collection for a practical plant breeding program that combines genomic and pedigree information. This core collection captures both, most of the current genetic variation of the breeding program and most of the future genetic variation based on crosses that were made. The core collection can be used to generate a haplotype reference panel for imputation methods, which allows for the cost-effective use of GBS methods on a large scale while still retaining the benefits of WGS.

## Results

### Genomic data curation

IStraw35 SNP array data curation resulted in 29,132 SNP calls for 891 unique genotypes. Twenty-nine additional genotypes were added to the SNP dataset by merging sequencing data with SNP array data. Pearson correlations between the SNPs in the sequencing data and the original SNP array were calculated for the genotypes that were present in both datasets. A total of 19,047 SNPs (out of 29,132) had a correlation > 0.7 and from these, 15,180 SNPs were found on the same chromosome as reported for the strawberry consensus map [18]. These 15,180 SNPs were selected in both the SNP array and the sequencing data in order to acquire genomic information for 920 genotypes. Subsequent filtering on minor allele frequency (MAF) and genome representation by linkage disequilibrium (LD) resulted in 8633 SNPs that were used to calculate the genomic relationships among the 920 genotypes (Table 1).

**Table 1:** Numbers of SNPs available for numbers of genotypes per genomic data curation step

Step	SNPs selection	SNPs (n)	Genotypes (n)
**1**	Curated iStraw35 SNP array	29,132	891
**2**	Merge SNP array with resequencing data	15,180	920
**3**	Selecting SNPs with MAF ≥ 0.05 and missing data ≤ 10%	13,613	920
**4**	Filtering SNPs on LD	8633	920

### Manual selection

To avoid the use of selections that have not finished phenotypic selection in the strawberry breeding program we excluded the advanced selections. Instead, we selected commonly used crossing parents that were used 4 or more times over a period of three years. This resulted in 69 genotypes to be included in the core collection. In addition, 56 genotypes were selected because of their specific trait-based genetic variation (*e.g.,* genotypes with a specific resistance; Table 2).

**Table 2:** Number of genotypes selected in the manual selection stage

Manual selection	Count of genotypes (n)
Recent crossing parents	69
Specific genetic variation	56
**Total genotypes selected**	**125**

### Pedigree improvement by comparing relationships

**Fig. 1:**
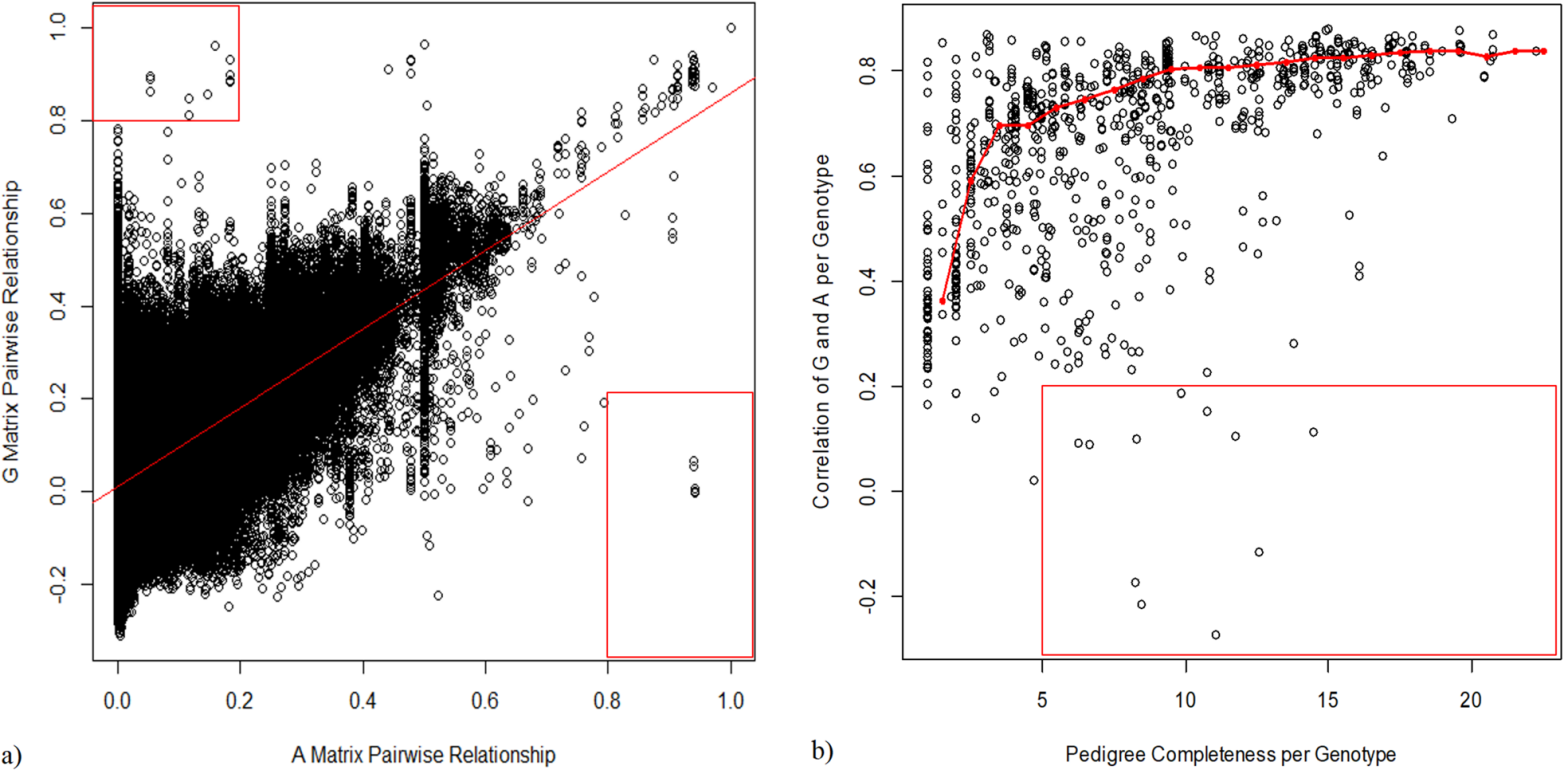
**a** Comparison of ${G}_{a}$ and *A*_*22*_. The red line shows a linear relation between the *G* and *A*_*22*_. **b** Plot showing correlation coefficients between ${G}_{a}$ and *A*_*22*_ per genotype on the y axis. The x axis shows the pedigree completeness (sum of kinship coefficients of all known parents per individual). The red line represents the moving median over an interval of 1 (pedigree completeness). Potential outliers are located in the red rectangular boxes

To estimate the accuracy of pedigree-based relationships, all pairwise combinations between ${A}_{22}$ and ${G}_{a}$ were plotted in Fig. 1a. The Pearson correlation coefficient between the coefficients in *A*_*22*_ and ${G}_{a}$ was 0.61. A few potential outliers were identified in this figure (highlighted in the red rectangular boxes) that either had high genomic-based relationship coefficients (> 0.8) and low pedigree-based relationship coefficients (< 0.2) or high pedigree-based relationship coefficients (> 0.8) and low genomic-based relationship coefficients (< 0.2).

To investigate how much the accuracy of *A*_*22*_ is influenced by the amount of pedigree information available, correlations between pedigree-based and genomic-based relationships were calculated per individual. These were plotted against the total sum of parental relationships per genotype. A clear trend can be seen between the sum of parent relationships and the correlation between the ${A}_{22}$ and ${G}_{a}$ matrices (Fig. 1b). The improvement of the accuracy of the pedigree relationship coefficient plateaus around or even before 10 generations (sum of total parental relationships) reaching a correlation of 0.8 (Fig. 1b). In addition to Fig. 1a, b was also used to identify outliers. Genotypes were identified as outliers (highlighted by the red rectangular box) if they have a low correlation (< 0.2) and high sum of parent relationship coefficients (> 5) and, for most of them, uncertainty was confirmed in either their pedigree records or in the genotyping (possible sample swaps). Several of these genotypes were also responsible for outliers in Fig. 1a.

### Minimum number of markers needed for accurate estimation of the *G* matrix

**Fig. 2:**
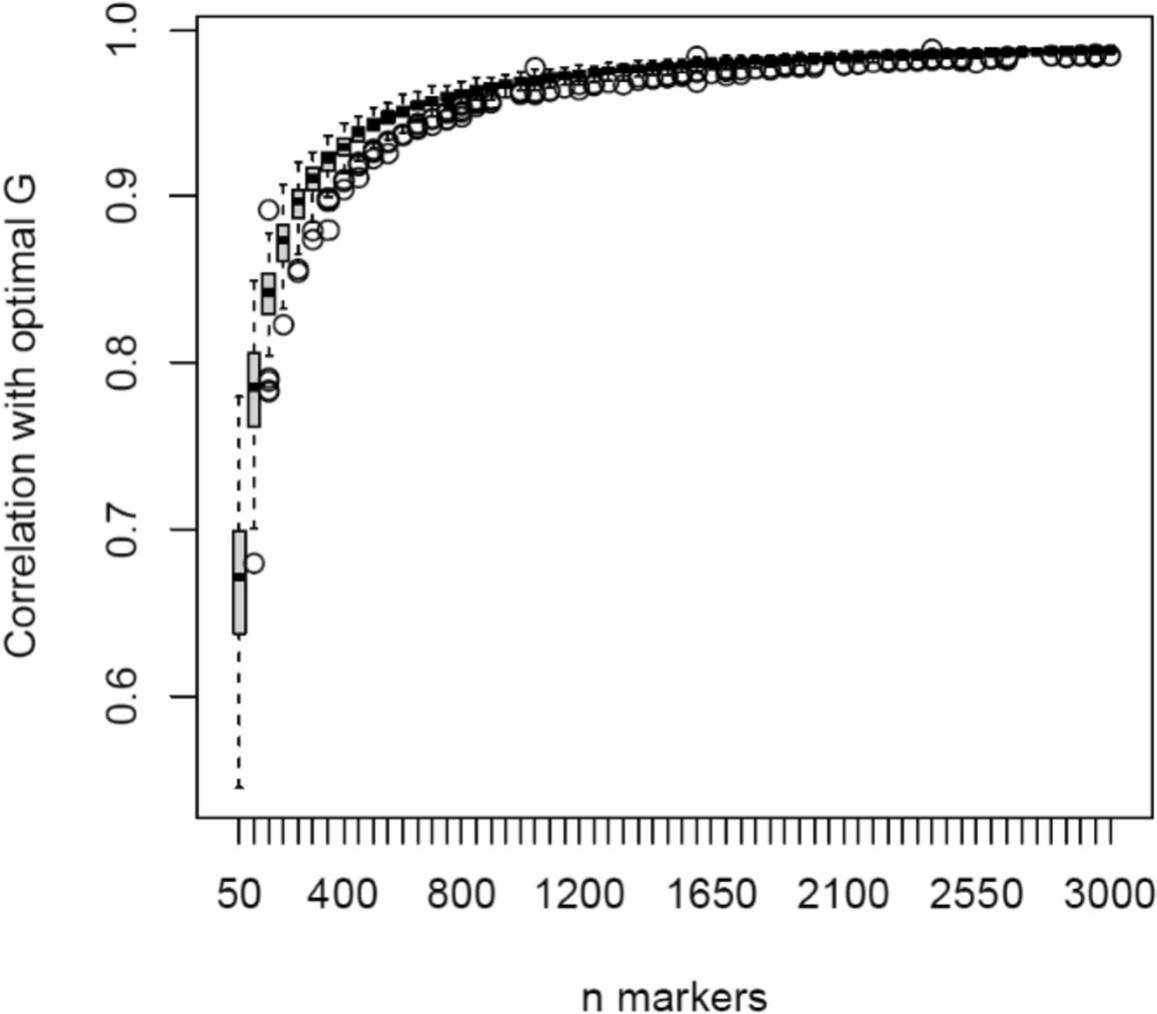
Subsampling analysis where correlations of optimal *G* matrix with G matrices based on varying numbers of SNPs are shown in boxplots

To estimate the number of markers needed to estimate the *G* matrix with high accuracy we performed a subsampling analysis, subsampling varying numbers of markers 200 times each. Pearson correlation coefficients between the *G* matrix, based on varying random subsets, and the optimal* G* matrix, based on the full set of 8,633 selected SNPs, were calculated (Fig. 2). The correlation increases exponentially when more SNPs are used for computation of genomic-based relationship coefficients. For more than 400 SNPs the correlation is higher than 0.9, with more than 850 SNPs the correlation is higher than 0.95; adding more SNPs leads to only marginal improvements. In addition, the variation in the correlation coefficients decreases with increasing marker numbers.

### Improved relationship coefficients for non-genotyped genotypes

To investigate the effect of combining pedigree and genomic information to estimate relationship coefficients among genotypes in our breeding program, we visualized the pedigree-based (*A*) and combined pedigree-genomic-based (*H*) relationship matrices for a representative subset of genotypes as heatmaps (Figure S1a) [19]. The genotypes are ordered according to the main strawberry types as described in Table 3. The same subset of genotypes is used in both Figure S1a, b. One of the differences between the two heatmaps is that the strawberry types can be better distinguished in Figure S1b. In addition, relationship coefficients in the pedigree-relationship matrix (Figure S1a) are generally lower than in the combined pedigree-genomic relationship matrix (Figure S1b) because of the presence of markers in the genomic-based relationships that are identical by state (IBS) not only due to being identical by descent (IBD) but also due to chance.

**Table 3:** Overview of characteristics of main strawberry types at Fresh Forward B.V

Strawberry types	Day neutral	Chilling requirement^a^
June bearing	No	High
Everbearing	Yes	High
Mediterranean	No	Low

^a^A low chilling requirement means that relatively few cold exposure hours are needed to get the plant out of winter dormancy

**Fig. 3:**
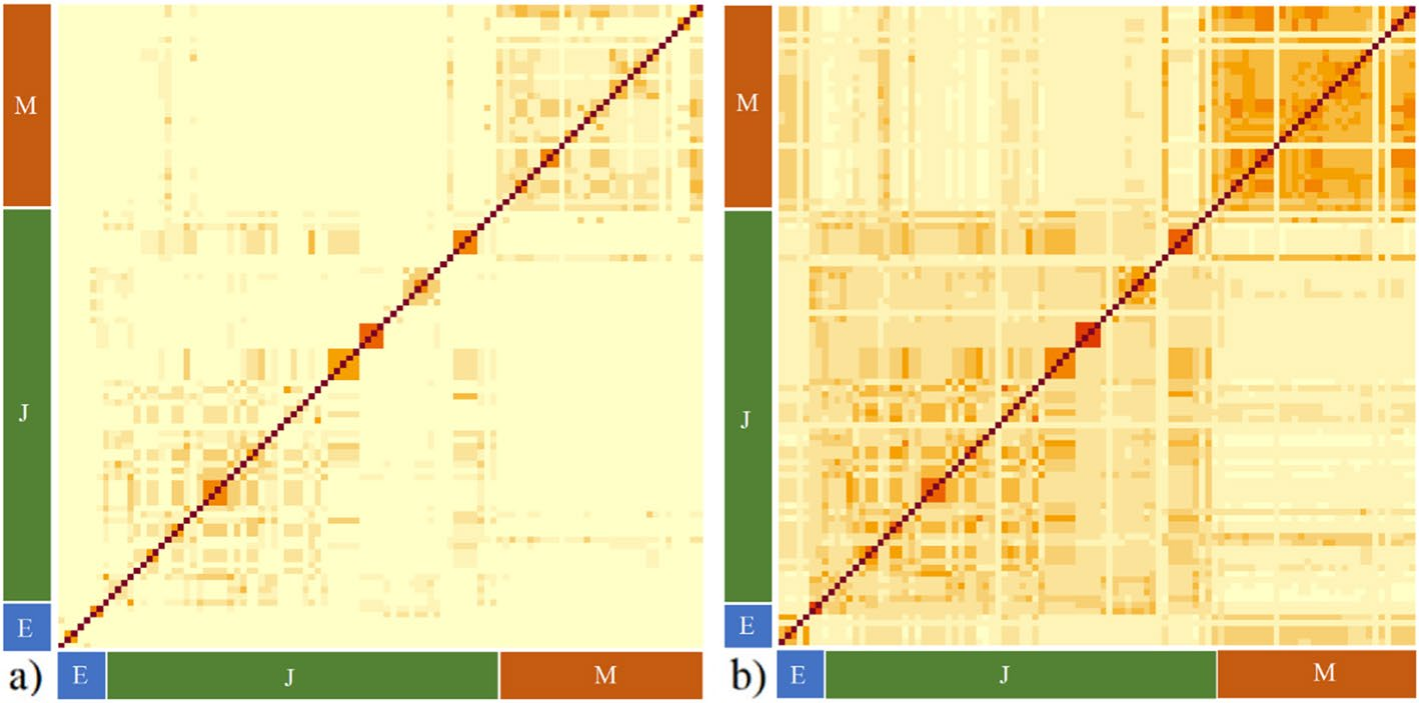
Heatmaps of relationship matrices. **a** Heatmap of genotypes without genotypic information of matrix *A*. **b** Heatmap of genotypes without genotypic information of matrix *H*. Scale is from little to no relationship (0; yellow) to a high relationship (1; red). Main strawberry types are shown: everbearing (E), June Bearing (J) and Mediterranean (M) types

To illustrate the influence of the genotyped individuals on the relationships of non-genotyped individuals in matrix *H*, we extracted all genotypes from Figure S1 that were not genotyped themselves. Figure 3a shows all pedigree-based relationships for this subset of ungenotyped individuals. Many are close to zero (in yellow) because there are no links among those genotypes in the pedigree records. Figure 3b visualizes the exact same genotypes but using the combined pedigree-genomic-based relationships. Most relationships that were (close to) zero in the pedigree-based relationship matrix are now higher and there is generally more contrast among genotypes. Most genotypes that have no known relationships in the pedigree-based heatmap do have estimated relationships in the pedigree-genomic heatmap. A few genotypes are present that still seem to have no relationships at all with other genotypes because they have no pedigree connections with any genotyped individuals (*e.g.,* they were introduced in the breeding program only recently).

The order of genotypes in these graphs is similar to those shown in Figure S1. In the bottom left corner only 5 genotypes that belong to the everbearing group are present. The next 57 genotypes are mainly June bearers and the genotypes in the top right corner are from the Mediterranean breeding program. A clear difference between Fig. 3a and b can be seen in the bottom right corner, where in the pedigree-based heatmap no relationships between everbearing genotypes and Mediterranean genotypes are present whereas in the pedigree-genomic-based heatmap these seem quite related. Also, some June bearing genotypes are more related to Mediterranean genotypes in the pedigree-genomic-based heatmap than in the pedigree-based heatmap. In addition, the relationships among the Mediterranean genotypes (block in the top right corner) are higher in the pedigree-genomic heatmap than in the pedigree-based heatmap.

### Finalized core collection by maximizing genetic variation

As can be seen in Figure S2, the A-NE first decreases exponentially but if the core collection is larger than ~ 100 the A-NE decreases linearly with every genotype added to the core collection. Therefore, we decided to select 192 genotypes for our core collection in addition to the genotypes that were already whole genome sequenced in previous experiments. This means that after the manual selection of 125 genotypes, 67 genotypes could still be added. As such, the core collection was finalized by adding 67 genotypes that were most often selected (from 3000 iterations) by optimizing the A-NE criterium [6, 14].

The iteration approach resulted in 59 candidate genotypes (out of 67 spots) where always the same genotype was selected in each of the 3000 iterations (Figure S2). The only uncertainty due to the stochastic algorithm of the Core Hunter package was for 8 candidate genotypes, where across the iterations, different genotypes have a similar influence in optimizing the A-NE criterium. For one of these eight, there were two alternative genotypes in different iterations, but one was selected in 35% of the cases whereas the other was selected in 65% of the cases and therefore we selected the latter one for the core collection. For each of six other spots, the 3000 runs resulted in approximately 50% of the cases for either one of two alternative genotypes, suggesting there was no difference between such a pair of genotypes in terms of A-NE minimalization of the core collection. Therefore, for each of the six spots the genotype that was preferred by the breeder was selected. Similarly, for the last spot (of the 8 spots), 4 different genotypes were selected in alternative iterations, each in approximately ¼ of the cases and the one preferred by the breeder was selected.

**Fig. 4:**
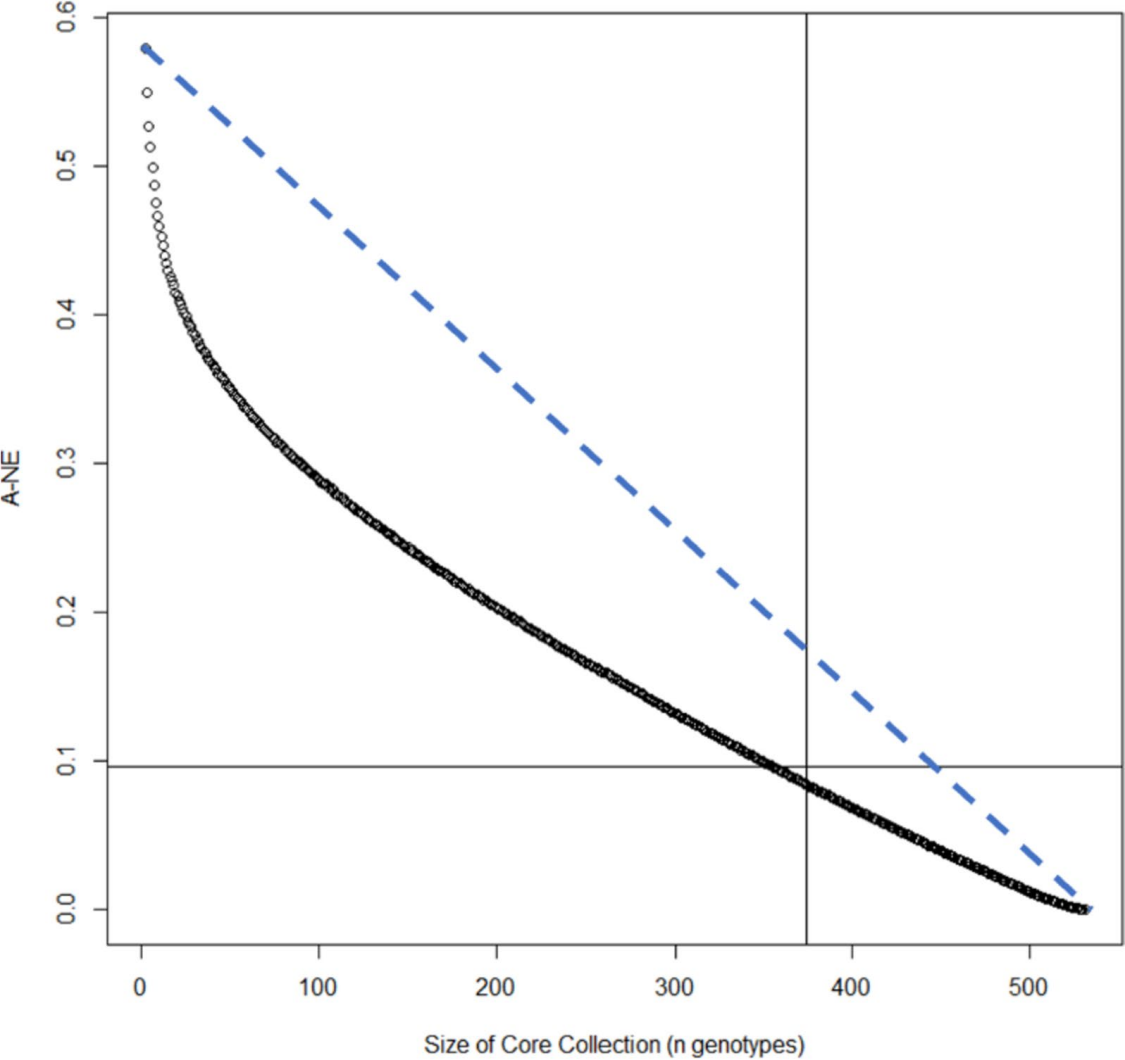
Minimized A-NE criterium with various sizes of a core collection. Vertical line is 371 genotypes (already sequenced + 192), and the horizontal line is the minimized A-NE criterium (0.096) of our core collection (including manual selection). The dashed diagonal line represents random sampling

This resulted in a core collection where the average distance of a genotype to an entry in the core collection had a minimum of 0.096 (A-NE criterium), while considering the genetic variation of the 125 manually selected genotypes and the genetic variation of all genotypes that already have been sequenced in previous experiments. This A-NE value of our core collection constructed by the manual selection (125 genotypes) followed by A-NE criterium optimization (67 genotypes) approaches the minimal theoretical A-NE value of a core collection that would be constructed by A-NE optimization only (Fig. 4). This means that 67 genotypes are sufficient to optimize the current genetic variation of the breeding program on top of the larger group of manually selected genotypes.

**Fig. 5:**
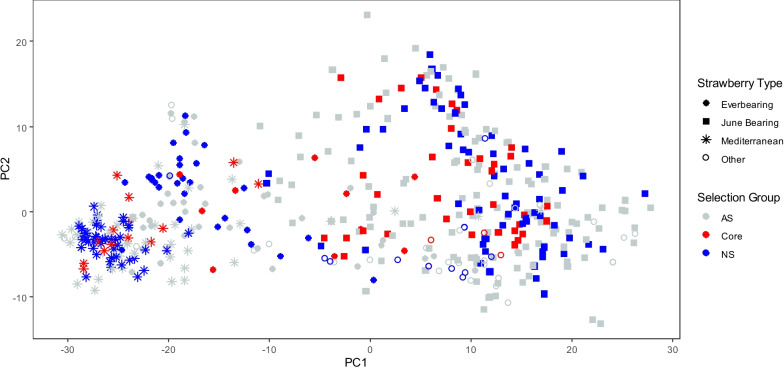
First 2 axes of a Principal Components Analysis (PCA) with all available genotypes and already sequenced genotypes (whole collection). The percentage of variance explained by PC1 and PC2 is 58% and 8%, respectively. In grey are all genotypes that were manually selected, all genotypes that were selected by the analysis of recent crossing parents and all genotypes that were previously sequenced (AS; Always Selected). In red are all genotypes selected by Core Hunter 3 (Core). Finally, all available genotypes that were never selected (NS) in any analysis are in blue

A principal components analysis of all relationships as computed in the pedigree-genomic-based relationship matrix (Fig. 5) shows the diversity of the breeding program. The first principal component axis explains most of the variation (58%) where it separates the sub-breeding programs such as June Bearing and Mediterranean genotypes from the everbearing genotypes.

### Distribution of subpopulations in the core collection

**Fig. 6:**
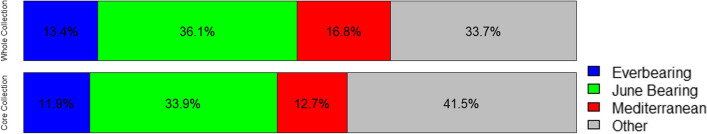
Distribution of subpopulations in the whole collection (top) and in the core collection (bottom)

The actual subpopulation representation in the breeding program at Fresh Forward (whole collection) has similar representation in the final constructed core collection (Fig. 6). In the core collection, the group “Other” is larger but this group is mainly composed of pre-breeding genotypes, *e.g.,* rare genotypes with specific traits, research populations and wild material and as such consists largely of manually selected material. The everbearing and June bearing groups approximately retained their relative size in the populations, the Mediterranean group is smaller in the core collection than in the whole collection.

## Discussion

Here, we introduce a stepwise approach for constructing a core collection in a commercial strawberry breeding program, combining (incomplete) pedigree records and SNP array data into a pedigree-genomic-based relationship matrix (*H* matrix) to guide the selection of a representative subset. We divided the approach into two parts: 1) First, we include the most frequently used crossing parents of all advanced selections that were still undergoing phenotypic selection, as well as “must-have” genotypes with specific traits that often represent diversity outside the breeding pool. 2) Second, the core collection was complemented with genotypes that maximized genetic variation within the collection. In gene banks, core collections are constructed by only performing the second step, which is often sufficient for their needs as their goal is to represent and conserve the genetic diversity of their collection [3, 4]. However, for a breeding program, which is a dynamic and evolving set of materials with continued selection, the goal of the core collection is to also represent the genetic variation to be released in the future. Therefore, our first step was the selection of genotypes that represent this future genetic variation.

### Selection of advanced selections

The first step, selection of genetic diversity that is present in advanced selections, which are still in the process of phenotypic selection, accounts for future genetic variation, because some of these advanced selections will become varieties or crossing parents in the coming years. The 69 selected parents have a degree of redundancy and overrepresentation, but this is not important because the main reason for choosing these parents is to include genotypes that are close to future advanced selections which makes genomic imputation easier for these future advanced selections [12, 20]. The use of core collection criteria (e.g., A-NE and E-NE) for this set is not desirable because these are aimed at avoiding redundancy and overrepresentation. Therefore, selecting genotypes based on counting the frequency of use as crossing parent is the simplest and best way of representing the variation present in advanced selections and thus important future variation within the breeding program.

### Extensive pedigree records decrease the need for genotyping

Nowadays, representative core collections are mainly constructed by only using genetic distances whereas pedigree-based relationships could be a supplement that allows decreasing the numbers of individuals that need to be genotyped. To investigate the accuracy of pedigree-based relationships in the breeding program, pedigree-based relationship coefficients were compared to genomic-based relationship coefficients. The Pearson correlation between *G*_*a*_ and *A*_*22*_ of 0.61 is somewhat lower than in several other studies where pedigree-based relationship coefficients showed correlations with genomic-based relationship coefficients of 0.73 to 0.85 [21–23]. However, these studies were all in animal breeding programs which generally have more accurate pedigree records and a smaller effective population size. This is different in plant breeding programs where genotypes can serve as both mothers and fathers and the effective population size can be large as neither the mother nor the father limits the number of offspring.

We found that the pedigree-based relationship coefficients were more accurate for genotypes with a more complete pedigree (Fig. 1b). However, inclusion of more than 10 generations of pedigree information is not needed to accurately estimate the pedigree relationship coefficient because the improvement by adding more pedigree information is only marginal (Fig. 1b). The correlations between pedigree-based and genomic-based relationship coefficients do not improve much anymore above a correlation of 0.8. Still, a perfect pedigree can only approach but never reach the accuracy of genomic-based relationship coefficients, for two reasons. First, pedigrees do not account for Mendelian sampling and can therefore not distinguish full sibs. However, for the construction of a core collection such detailed relationships are not needed because the aim is to capture most of the genetic variation present in a (future) breeding population rather than variation among full sibs. Second, genotypes without a known common ancestor are assumed to be unrelated in pedigrees whereas they are always somewhat related (*i.e*., there will always be a common ancestor if you go back far enough). These relationships are important for the construction of a core collection because they heavily influence relationships of their offspring with offspring of other genotypes.

We also identified outliers based on both Fig. 1a and b, where the pedigree records do not match the genotypic information of the genotypes. This can be caused by sample swaps and missing or incorrect pedigree records. These outliers are targets for further investigation to check whether pedigree modifications can be proposed, for example by using the method described by Endelman et al. [24].

### Pedigree-genomic-based relationships

Although pedigree-based relationship coefficients are accurate, several limitations exist such as missing links in the pedigree, incomplete pedigree records and pedigree errors. To overcome these limitations and still omitting the need to genotype the whole population, we computed pedigree-genomic-based relationships [17, 25]. We showed that using a hybrid pedigree-genomic relationship matrix can overcome incomplete pedigree records (Figure S1). This method can also correct for missing links, relatedness among founders (genotypes that have at least one unknown parent in the pedigree) and mistakes in the pedigree, resulting in more accurate relationship estimates for non-genotyped genotypes [26]. For instance, direct crosses between everbearing and Mediterranean genotypes are rarely made (no pedigree relationships) but the everbearing type originated, in 1980, from the Californian breeding population which are Mediterranean genotypes, so we can still expect relationships among the everbearing and Mediterranean genotypes [27]. The pedigree-genomic-based relationships of only non-genotyped genotypes revealed such relationships between the everbearing and Mediterranean strawberry types which could not have been found based on pedigree-based relationships solely. However, the hybrid matrix can only correct for these kinds of issues if genotypes that have these pedigree issues have a (in)direct connection in the pedigree records with genotypes that are genotyped. Therefore, it can be advantageous to genotype specific genotypes to optimally utilize the power of the pedigree-genomic-based relationship approach. The most important genotypes to be included are the pedigree’s founders that have a major impact on the breeding program. If, for practical reasons, these founders are not available anymore (e.g., not propagated anymore), closely related genotypes are a suitable alternative.

### Influence of marker number on reliability of relationship estimates

From our results (Fig. 2), we derive that only 400 SNPs are already enough to reliably compute genomic-based relationship coefficients among strawberry genotypes. This is in line with studies on genomic prediction, where limited impact of reducing the number of markers on prediction accuracies are observed [28, 29]. A major advantage of these low numbers of SNPs is that extensive genotyping methods like SNP arrays or WGS can be replaced by more high-throughput GBS techniques, *e.g.*, AmpliSeq, targeted SNPseq, Single Primer Enrichment Technology (SPET) or Molecular Inversion Probe (MIP) sequencing [30–32]. By employing one of these cost-effective genotyping approaches, new core collections can be developed based on more accurate pedigree-genomic-based relationships because more founders (or their relatives) can be genotyped.

### Final core collection

To complement the selection of the most frequently used parents of advanced selections and rare genotypes, additional genotypes were selected that minimized the average distance of genotypes in the whole collection to the closest genotype in the core collection by exploiting the pedigree-genomic-based relationship matrix. This final core collection then is a subset of genotypes that contain most of the genetic diversity that will become important in the future (parents of advanced selections and rare specific genotypes) but that also has maximum representation of the genetic variation in the current breeding program. For example, the PCA plot indicated that the Mediterranean strawberry types were less diverse than the June bearing types (Fig. 5). This suggests a smaller genetic basis of the Mediterranean types which was also mentioned in other studies [25, 33]. This smaller genetic basis is reflected in the core collection in the sense that relatively fewer genotypes were selected from this strawberry type than from the other types (Fig. 6).

### Future considerations

As this core collection captures most of the current and future variation of the breeding program, we expect that this core collection only needs to be updated when new alleles are added to the breeding program. In addition, if this core collection is going to be used for imputation purposes, it is important to check every few generations if the imputation is still accurate, because the distance between the core collection and the to-be-imputed genotypes increases by every cross that is made. If the imputation accuracy starts to decline, recent crossing parents should be added to the core collection.

## Conclusions

In this study we explored the construction and use of a core collection in a commercial plant breeding program. Traditionally, core collections are used by gene banks to obtain a wide diversity of genetic variation of their germplasm in a small subset of representative genotypes based on genomic relationships. Here we employ a stepwise approach to obtain a core collection that is representative of future genetic variation to be used as reference panel for cost-effective GBS methods. This approach is a useful tool for adopting GBS methods by commercial plant breeding programs and utilizes both genomic and pedigree data often available within such programs.

## Methods

### Materials

The strawberry breeding program at Fresh Forward B.V. consists of three general types: June bearing, everbearing and Mediterranean strawberry types. They can be distinguished by the difference in being day neutral, or by their chilling requirement (Table 3) [34]. We define everbearing strawberry types by the presence of the main locus (*FaPFRU*) for day neutrality [35]. The chilling requirement is determined by the strawberry genotype’s performance in a low and a high chilling environment.

In terms of selection stages, the breeding program (including the pre-breeding program) consists of varieties, crossing parents, advanced selections (that have not yet completed all the selection cycles) and genotypes used for specific traits. In this study we only considered *F. x ananassa* germplasm.

### Genomic information

SNP array data was available for 891 genotypes, which included 29,132 high-quality SNPs [36]. Most of the 891 genotypes are from the current in-house breeding program. Resequencing data (Illumina paired-end) was used to increase the number of genotypes with genomic data. To get an integrated set of SNPs and individuals that are either genotyped by the iStraw35 SNP array or by re-sequencing, the probes of the iStraw35 SNP array were aligned on the *Camarosa v1* genome [37]. Then, Pearson correlation coefficients between the SNP array calls and the dosage calls of the resequencing data for 119 overlapping genotypes were calculated. All found SNPs that have a squared correlation coefficient ≥ 0.7 and which were on the same subgenome on the strawberry consensus map [18] were retained. The selected SNPs were then curated by removing all SNPs that had more than 10% missing data and all SNPs with minor allele frequencies (MAF) < 0.05. Missing dosages were imputed by the mean SNP dosages to minimize their influence in further steps. Additionally, linkage disequilibrium (LD)-based pruning was applied in order to get independently segregating SNPs for downstream analyses [25].

### Manual selection

Before we employed *Core Hunter 3* to obtain a core collection, we manually selected advanced selections and specific genotypes because they represent important future genetic variation. The genetic variation currently present in the breeding program is different from the future genetic variation due to the introduction of new genotypes and the selection pressure which changes allele frequencies over time. This means that the current breeding program does not fully represent the genetic variation of the future breeding programs. However, a large part of future genetic variation is already present in advanced selections and specific genotypes. Advanced selections cannot be selected by optimizing the A-NE criterium because of two reasons: First, optimizing the A-NE criterium will give too much weight to the advanced selections in comparison to other genotypes because they include many siblings. As a result, optimizing the A-NE criterium tends to select too many genotypes that are related to these sibling families because they will minimize the average distance of a genotype to the nearest entry (A-NE) in the core collection the most [6]. Second, the selection process of these advanced selections is still ongoing at this stage, and it is unknown which will still be discarded, and which will become cultivars or crossing parents. Alternatively, the genetic variation of the advanced selections is best selected by choosing the parents that are most often used as parents for these advanced selections in recent years. This ensures that their genetic variation is maximized and that the parents that have the most potential for the future according to the breeders are selected. Therefore, to include most of the genetic variation of these advanced selections, crossing parents were selected that were used 4 or more times in crossings over the last 3 years (2020, 2021 and 2022).

In addition to the advanced selections, some genotypes possess specific alleles for certain traits that will become more abundant in the future breeding program. For instance, genetic variation of genotypes that have a specific disease resistance or genotypes with extreme traits (*e.g.,* in strawberry this may entail extremely firm or extremely well tasting fruits) that are of particular interest to breeders. These specific genotypes are “must haves” for the core collection and we do not want to rely on the A-NE criterium for the selection of these because these are not representative genotypes for the current breeding program and will not be selected. Instead, these specific genotypes can easily be identified by experienced (pre) breeders who work with them. Therefore, we manually selected several specific genotypes to make sure that they are part of the core collection.

### Relationships among genotypes

#### Pedigree relationships

All pedigree records of the strawberry breeding program at Fresh Forward B.V. spanning selections originating from 1930–2022 were used to compute the pedigree-based relationship coefficient matrix (*A*). The *A* matrix was estimated using *create.pedigree* and *kin* from the package *synbreed* [19]. The pedigree-based relationship coefficient of genotype A and B was calculated as follows:

$$0.5*[\left({A}_{1}, {B}_{1}\right)+\left({A}_{1}, {B}_{2}\right)+\left({A}_{2}, {B}_{1}\right)+\left({A}_{2}, {B}_{2}\right)]$$

where A_1_ and A_2_ are the alleles from genotype A and B1 and B2 are the alleles of genotype B. (A1, B1) denotes the probability that A1 and B1 are identical by descent (*IBD*).

#### Genetic relationships

Genomic-based relationship coefficients were estimated among all genotypes on the curated set of SNPs. The genomic-based relationship coefficient matrix (*G* matrix) was calculated following the VanRaden method with the function *A.mat* of the R-package *rrBLUP* [38, 39].

### Construction of the H-matrix

As described in Legarra et al. [17] a hybrid pedigree-genomic relationship matrix (*H*) was estimated for the entire pedigree: Here, matrix *A* consists of 4 different submatrices (*A*_*11*_*, A*_*12*_*, A*_*21*_*, A*_*22*_), where subscript 1 denotes ungenotyped and subscript 2 denotes genotyped individuals. So, for example, *A*_*12*_ are the pedigree-based relationships of non-genotyped individuals with genotyped individuals and *A*_*22*_ are the pedigree-based relationships of all genotyped individuals among each other. So, *A*_*22*_ is the same as the *G* matrix, only the relationship coefficients are estimated with pedigree information instead of the SNP data. While both *A*_*22*_ and *G* yield equivalent relationship estimations, an offset in scale exists. Therefore, to obtain compatible matrices, *G* was scaled to *G*_*a*_, where:

$${G}_{a}=\beta G+ \alpha$$

to guarantee that the average of diagonal elements of *A*_*22*_ equals the average of the diagonal elements of *G* and the average of all elements of *G* equals the average of all elements of *A*_*22*_ [26]. Finally, the hybrid (*H*) matrix was computed following the method described by Legarra et al. [17], using the following formula.

$$H= \left[\begin{array}{cc}{A}_{11}+{A}_{12}{A}_{22}^{-1}\left({G}_{a}-{A}_{22}\right){A}_{22}^{-1}{A}_{21}& {A}_{12}{A}_{22}^{-1}{G}_{a}\\ {G}_{a}{A}_{22}^{-1}{A}_{21}& {G}_{a}\end{array}\right]$$

### Estimating accuracy of pedigree-based relationships

#### Accuracy of matrix *A* compared to matrix G

To estimate the accuracy of the A matrix, the Pearson correlation coefficient was calculated between ${A}_{22}$ and ${G}_{a}$. In addition, to gain insight into the accuracy of pedigree-based relationship coefficients per single genotype, Pearson correlation coefficients were calculated between all pedigree-based relationship coefficients of a single genotype (a single column in ${A}_{22}$) and all its genomic-based relationship coefficients (a single column in ${G}_{a}$), resulting in a Pearson correlation coefficient for each genotype in ${G}_{a}$.

#### Pedigree completeness estimation per individual by using a network analysis approach

To estimate the minimum amount of pedigree information per genotype (pedigree completeness) needed to accurately estimate pedigree-based relationship coefficients, we estimated the pedigree completeness (amount of pedigree information per individual). In principle, a more complete pedigree will lead to more accurate relationship estimates among genotypes. However, distant ancestors have very little impact on the estimation of relationships among genotypes [40]. To consider the declining impact of more distant ancestors to the estimation of relationship coefficients, we used the pedigree-based relationship coefficient between a genotype and its ancestor as measure of the impact the ancestor has on the estimation of all relationships of that genotype. As a result, the sum of total pedigree-based relationships of a genotype’s ancestors is considered as a measure of pedigree completeness per genotype. In theory, without inbreeding, this pedigree completeness could be interpreted as number of ancestral generations in the pedigree per genotype (total theoretical pedigree relationships per generation is equal to 1, e.g.: direct parents = 2 times 0.5 and grandparents = 4 times 0.25).

To calculate the pedigree completeness, we modified matrix *A* into matrix A* where we changed all relationships of parents with offspring to 0 and kept all relationships of offspring with their (grand) parents (their ancestors). From matrix A*, we computed the pedigree completeness per genotype by computing the sum of total pedigree-based relationships of a genotype’s ancestors per single genotype.

The pedigree completeness per single genotype was then plotted against the correlation between pedigree-based and genomic-based relationship coefficients. Genomic-based relationship coefficients are considered to be more accurate than pedigree-based relationship coefficients and therefore the correlation coefficient between these two are a good indicator for the accuracy of pedigree-based relationship coefficients [16]. To obtain these correlations, the Pearson correlation coefficient was computed per single genotype between its genomic-based relationship coefficients and its pedigree-based relationship coefficients.

### Final selection representative core collection

The A-NE criterium is applied to genotypes that are not in the process of phenotypic selection anymore because these genotypes are representative of the current state of the breeding program. For these, we can maximize the genetic variation of the full breeding program in the core collection by minimizing the A-NE criterium [6]. *Core Hunter 3* is a core collection selection tool which can use one or several distance metrics [14]. The package has implemented A-NE and other core collection criteria as defined by Odong et al. [6].

For being able to use both genomic-based relationships and pedigree-based relationships in distance-based methods we scaled the pedigree-genomic-based relationship matrix [6]. In this way, we can use all advantages of the combined pedigree-genomic-based relationship matrix in distance-based methods such as optimizing the A-NE criterium. To obtain a matrix that can be used in *Core Hunter 3* from this pedigree-genomic-based correlation matrix (${H}_{cor}$), we first converted the covariance matrix *H* into a correlation matrix (${H}_{cor}$) with diagonal values equal to 1. Subsequently, we scaled the correlation matrix (${H}_{cor}$) using the following equation in a custom R script:

$$D=\frac{\left(1-{H}_{cor}\right)+|\mathrm{min}({H}_{cor})|}{1+|\mathrm{min}({H}_{cor})|}$$

where ${H}_{cor}$ is the correlation matrix of covariance matrix *H* and *D* is the scaled matrix of similar size as ${H}_{cor}$ where all diagonal values are equal to 0 and all other values are between 0 and 1.

The obtained scaled relationship matrix (*D*) was then filtered because core collection computation by optimizing A-NE is influenced by the definition of the whole collection, as the CC-I criteria will optimize the average relationship of each genotype of this whole collection to the closest entry in the core collection. This whole collection is defined as all genotypes that were propagated in-house in 2022, including varieties, crossing parents and specific genotypes but without advanced selections to avoid redundancy and overrepresentation in the final core collection. The scaled relationships of all genotypes of the whole collection were used to obtain a CC-I collection by using the *sampleCore* function from the *Core Hunter 3* package considering all specific genotypes, all most used recent crossing parents of advanced selections and the genotypes that had already been sequenced in previous experiments. The computation of a CC-I core collection was iterated 3000 times because of the stochastic algorithms used in the *Core Hunter 3* package and the best consensus core collection was selected [6, 14].

To investigate the minimum size of our stepwise constructed core collection and to check its representation of the current genetic variation in comparison to a core collection that is only constructed by A-NE optimization, we calculated the optimized A-NE values for varying core collection sizes.

### Relationships between number of markers and accurateness of the *G* matrix

A valid question is how many SNPs are needed to compute a reliable genomic-based coefficient matrix. Therefore, we performed a subsampling analysis where varying subsets of random markers were selected to compute a genomic-based relationship matrix (*G*_*s*_; [39]). The subset size varied from 50 to 3000 randomly selected markers with increments of 50. For each subset the genomic-based relationship matrix (*G*_*s*_) was computed and the Pearson correlation coefficient between this *G*_*s*_ matrix and the optimum* G* matrix (based on the markers that resulted from the genomic data curation; step 4 in Table 1) was calculated. Per subset size, 200 iterations were performed. The minimum number of markers needed for accurate estimation of genomic-based relationships was estimated by checking how large a random subsample needs to be to have a minimum correlation of ≥ 0.95 with the optimum genomic-based relationships (*G*).

### Supplementary Information

**Additional file 1: Figure S1.** a) Heatmap of matrix A of all genotypes of the ‘Whole Collection’ b) Heatmap of matrix H of all genotypes that are in the ‘Whole Collection’. Scale is from little to no relationship (0; yellow) to a high relationship (1; red). Main strawberry types are shown: everbearing (E), June Bearing (J) and Mediterranean (M) types. **Figure S2.** Occurrence of genotypes in 3000 repeated iterations for complementation of the core collection of 67 genotypes.

## Data Availability

R scripts are available from the corresponding author on reasonable request.
