# Supplementary material for: Construction of a strawberry breeding core collection to capture and exploit genetic variation
Source: BMC Genomics. 2023 Dec 5;24:740. doi: 10.1186/s12864-023-09824-1 (PMC10696700; doi:10.1186/s12864-023-09824-1)
Supplement: Supplementary file 1 — Additional file 1: Figure S1. a) Heatmap of matrix A of all genotypes of the ‘Whole Collection’ b) Heatmap of matrix H of all genotypes that are in the ‘Whole Collection’. Scale is from little to no relationship (0; yellow) to a high relationship (1; red). Main strawberry types are shown: everbearing (E), June Bearing (J) and Mediterranean (M) types. Figure S2. Occurrence of genotypes in 3000 repeated iterations for complementation of the core collection of 67 genotypes. [file 12864_2023_9824_MOESM1_ESM.pdf]

SUPPLEMENTARY FIGURES

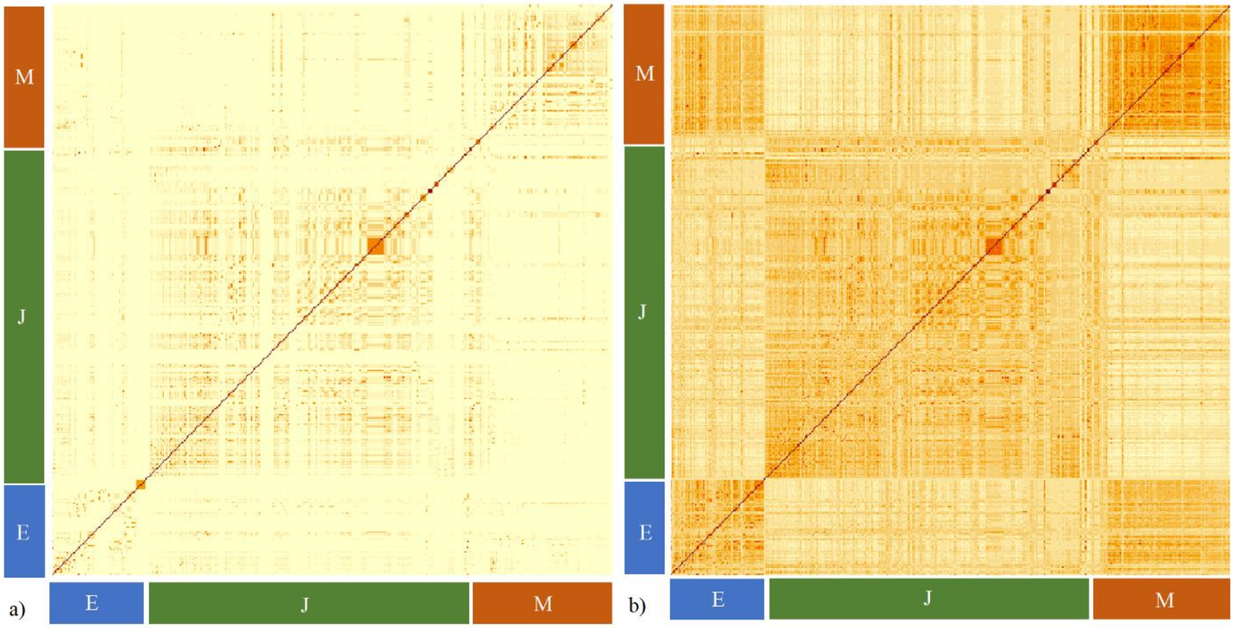

Figure S 1. a) Heatmap of matrix A of all genotypes of the 'Whole Collection' b) Heatmap of matrix H of all genotypes that are in the 'Whole Collection'. Scale is from little to no relationship (0; yellow) to a high relationship (1; red). Main strawberry types are shown: everbearing (E), June Bearing (J) and Mediterranean (M) types.

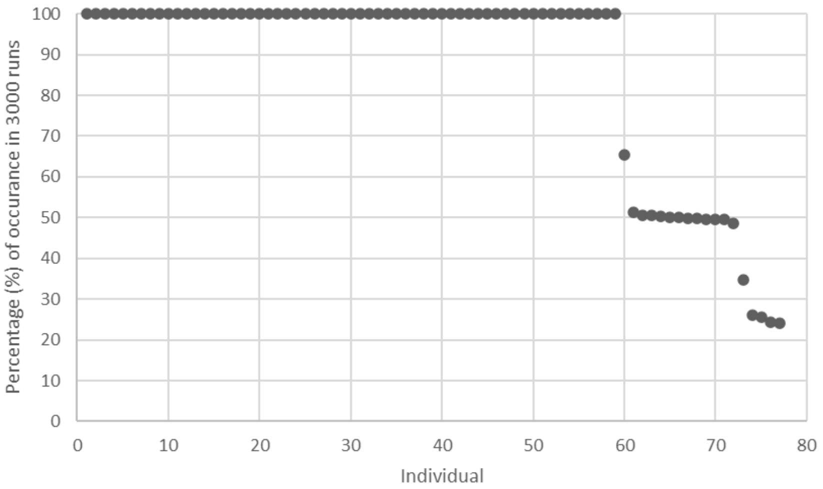

Figure S 2. Occurrence of genotypes in 3000 repeated iterations for complementation of the core collection of 67 genotypes.
